# Relationship Between Postnatal Depression of Mental Health Patients and the Psychological Health of Their Offspring

**DOI:** 10.3389/fpsyt.2021.772744

**Published:** 2022-01-03

**Authors:** Fairuz Nazri Abd Rahman, Yun Yaw Wong, Ahmad Qabil Khalib

**Affiliations:** ^1^Faculty of Medicine, National University of Malaysia, Bangi, Malaysia; ^2^Ministry of Health (Malaysia), Putrajaya, Malaysia

**Keywords:** postnatal depression, psychological health, offspring, psychiatric patients, children

## Abstract

Postnatal depression is a major illness affecting maternal and family health. The rate of postnatal depression among mental health clients is postulated to be higher than in the community due to the added brain assault. Children of parents who are mental health clients are more likely to have psychological problems compared to children from other parents in the community. This study investigates the rate of postnatal depression among mental health clients and their offspring's psychological health. A total of 140 mental health clients were assessed using the Edinburgh Postnatal Depression Scale (EPDS). They subsequently completed the Strength and Difficulties Questionnaire (SDQ) regarding their children. The majority ethnicity was the Kadazan (40.7%). The mean age of mothers was 38.6 (7) years with most having a secondary education (53.6%) and a household income per month of < RM1000 per month (27.1%). The postnatal depression rate was 47.8%. Higher EPDS scores were associated with higher total SDQ scores in their offspring. Model 1 was unadjusted, giving an OR of 5.65 [95% CI (3.74, 7.55)], *p* < 0.001. After adjustment for confounders, Model 2 had an OR of 5.51 [95% CI (3.57, 7.46)], *p* < 0.001. More efforts need to be given to the early detection of maternal depression and its prompt treatment in mental health clients because of the relationship with the psychological health of the offspring.

## Introduction

Postnatal depression is often defined as depression commencing within 6 weeks postpartum (1). A recent meta-analysis found that the incidence of depression during the postnatal period is about 9.8% in mothers in the community (2). In Malaysia, the postnatal depression rate was found to be 4.4% (3). Whereas, the prevalence of mental health issues in general in mothers was 22.3% (4). It is already known that motherhood itself is a stressful experience, what more if the mother suffers from mental illness, the problem faced by mothers is then compounded. Among the themes that are associated with mothers with mental illness are the nature of the changing diagnoses, of stigma by society, custody issues where mothers with mental illness lose custody of their children, family support and single parenthood (5). They also worry about societal perception of their children as offspring of mothers who have mental illness (6).

Depression commonly affects patients with mental health issues. The lifetime prevalence of depression in the community is 10.8% according to one meta-analysis (7). Depression is a common comorbidity in many psychiatric disorders, the most common being social anxiety disorder (31.3%), generalized anxiety disorder (23.6%), post-traumatic stress disorder (PTSD) (20.6%), and obsessive-compulsive disorder (14.3%) (8). How does depression develop in psychiatric disorders? Psychosis, as an example, is thought to be a major life event, and is depressogenic especially if the patient appraises the event as a loss or humiliation (9). It is not surprising then that the same hypothesis may apply to any experienced mental disorder in terms of the development of depression. New studies have shown an existing mental disorder is a risk factor to develop another mental disorder (10), and this relationship was found to be bidirectional (11). The rate of postnatal depression among mental health clients is postulated to be higher than in the community due to the added assault to the brain. Chronic stress predisposes to depression and may be caused by structural degeneration of the prefrontal cortex and hippocampus which later leads to impaired emotion regulation and cognitive impairment (12).

Mothers in most cultures and circumstances spend the bulk of the time raising their children, and traditionally children have the most contact time with their mothers. It was found that children with a parent who had presented to the mental health services are 2–5 times more likely to score higher on the SDQ compared to children from normal parents (13). This has long term implications continuing into adolescence and adulthood. For example, children of mothers with affective disorders were found to have higher rates of anxiety disorders, depressive disorders, conduct disorders and substance use (14). In another study, children of mothers with persistent high levels of depressive symptoms had lower IQ scores (15, 16). The mechanism to which this happens can be explained by mothers with depression being less sensitive to their children's needs (17, 18). There are also mothers who were more hostile and impatient toward their children (19). Changes in maternal behavior have been found to mediate the association between maternal depression and toddlers' behavioral problems (20). Another hypothesis posits that depressive symptoms in the mother increases the likelihood of marital conflict (21, 22). Marital conflict within the family in turn affects the sense of security of the child and her development (23). When emotional security is affected, the child tries to regain it within the family. This may be attempted by avoidance or exhibiting behaviors to distract parents in hopes to stop the conflict, thus leading to the various emotional or behavioral problems in the child (24).

The studies quoted so far are different to the cultures being studied in this paper. To our knowledge, there was a study in peninsular Malaysia investigating the emotional and behavioral problems in adolescent offspring of mothers with depression (25). The mentioned study showed offspring of mothers with depression had more emotional and behavioral problems, more externalizing, more aggressive behavior as well as attention problems. Few studies refer specifically to the populations in Borneo Malaysia. Sabah is known as the poorest state with the highest population of non-citizens. Constituting one-third of the indigenous population in Sabah, Kazadans are among the prominent indigenous groups (24.5%) in the state of Sabah, Malaysia (26). Exposure to Christianity, western education, and the Malaysian government's effort to modernize the nation have contributed to making the Kadazans the most urbanized among all of the indigenous groups in Sabah (27). Traditionally, the well-being of the mother during pregnancy and delivery is placed in the hands of the midwife (28). Many taboos are observed during pregnancy. Amulets are worn to ward off disturbances from evil spirits. The name of the baby will be chosen by the grandparents. Like other Malaysian communities, Kadazan mothers exceeded fathers in the amount of time they spent in cleaning, feeding, and playing with infants and in their levels of engagement in direct care of infants (27). Similarly, for the Murut and the predominantly Muslim Bajau, pregnancy taboos and birth customs are practiced, with the midwife playing a central part of the care. This includes prenatal massage. The death of a baby was believed to be due to witchcraft or violation of taboos. Non sterile practices such as using bamboo to cut the umbilical cord were used where access to modern medical care was limited in remote areas of Sabah (29). Neonatal mortality rate in Sabah is still the highest among the Malaysian states at 5.4 deaths per 1,000 live births in 2019. Naturally, access to modern medical care is a concern for non-citizens. As for the Malay and Chinese communities, the women undergo a confinement period of 30–44 days after delivery accompanied by a confinement nanny. This is the time when they undergo hot compress, herbal baths, body wraps and body massages to recover from delivery (30). Mohamad Yusuff (31) and her colleagues assessed 2,072 Sabahan women in 2009–2010 and found the prevalence rate of postnatal depression to be 14.3%. A computerized birthing system for Sabah was set up in 2012 (32) to record high risk pregnancies, births and immunizations, but there is no mention of assessment for mental health issues.

The Malaysian population, as compared to the Western population are influenced by conditions of poorer economic development, different cultures, and local practices which may have different results from any previously done studies, which mainly focused on populations from developed countries. This study might shed some light on the effects of postnatal depression in mental health clients on the psychological well-being of their offspring in the Malaysian population.

## Methods

### Setting and Population

This is a cross sectional study of 140 female mental health clients conducted at the outpatient clinic and inpatient wards of Hospital Mesra Bukit Padang, Sabah, Malaysia between 15 June 2020 and 15 December 2020. Hospital Mesra Bukit Padang is one of the largest psychiatric referral centers in Borneo Malaysia serving a multicultural population of various races. It has ~300 beds.

Sample size calculation for cross sectional studies/surveys for quantitative variables uses the formula *N* = Z2P(1-P)/d2 (33). The prevalence from a previous study is 9.8% (2). With the precision set as 0.05, thus the sample size *n* = 1.962 × [0.098 (1–0.098)/0.052] = 136. After addition of an estimated 20% for non-respondents, an additional 28 participants were added, making the final target sample size = 164.

Convenience sampling was used. Mothers using the services in Hospital Mesra Bukit Padang who have at least one child in the age group of 4–17 years old were asked to fill in once only regarding the child with the most behavioral & emotional problems as well as their depressive symptoms, if any, during any pregnancy. All questionnaires were self-rated. Exclusion criteria were inability to read and answer the questionnaires and those who could not understand or speak the languages used in the study (English or Malay). All mothers provided written consent.

### Maternal Depressive Symptoms

Maternal depressive symptoms were assessed using the EPDS (34). Although originally designed to assess postpartum depressive symptoms in the past 7 days postpartum, it has been found to be a valid measure of depressive symptomatology in parents beyond the immediate postpartum period (35). Thorpe (35) studied parents with children up to the age of 22 months. In a more recent study, Lee et al. (36) studied mothers with children up to the age of 48 months. In the Avon Longitudinal Study of Parents and Children (ALSPAC) (37), a currently ongoing birth cohort, parents have been assessed using EPDS at time points up to the period of 11 years and planned to be assessed at 18 years because of the longitudinal nature of depression. In this study, mothers were required to recall postnatal depression symptoms of any previous pregnancy within 6 months of delivery. The questionnaire is a 10-item scale and answers are on a 4-point Likert scale from 0 to 3 for a possible range of 0–30, where a higher score indicates a higher level of depressive symptoms. The EPDS has been validated in the Malay language (38). The EPDS is not used commonly as a diagnostic scale. However, if a cut off score of ≥13 is used, it is an indication of clinically significant depression (39). The EPDS has a 59.5% sensitivity and 88.4% specificity for major depressive disorder if the score is ≥13 (40).

### Child Psychological Outcomes

The SDQ (41) is a scale to assess behavior and emotions of children. It has recently been validated in the Malay language (42). The version of SDQ used is a parent rated questionnaire designed for children from ages 4–17 years. There are 25 items in this scale with scoring of 0 (not true), 1 (somewhat true) and 2 (certainly true). The scale has 5 subscales namely emotional symptoms, conduct problems, symptoms of hyperactivity/inattention, peer relationship problems, and prosocial behavior. The subscales except prosocial behavior are added up to form a total difficulties score. The SDQ has a sensitivity of 62% and the specificity was 83% for total difficulties score (43).

## Results

The distribution of demographics and risk factors are presented in Table 1. A total of 147 mothers were approached initially but 7 did not consent to join the study. A total of 140 mothers participated in our study. The mean age for the participants was 38.6 (7) years. The Kadazan race made up 40.7% of participants. The Bajau made up of 16.4%, followed by the Chinese 12.9% and other races made up 17.9% of the participants. The Murut, amongst the most populous race in Sabah only recorded 1.4%. Islam was the most professed religion, comprising 53.6% and then Christians at 41.4%. The group with the least representation identified as agnostics at 0.7%. The proportion of the makeup in each subgroup for ethnicity and religion is fairly similar when EPDS positive and negative were compared. Single mothers comprised of 5.7% overall. There were more mothers who were married in the EPDS positive group at 85.1%. There were more mothers who were divorcees or widows in the EPDS negative group at 15.1%. In this study, 32.9% of participants had a diagnosis of schizophrenia, 27.1% had depressive disorders, 20% had bipolar disorder, 12.1% had anxiety disorders, 0.7% had schizoaffective disorder, and 7.1% for other diagnoses. There were more affective disorders in the EPDS positive group comprising bipolar disorders and depressive disorders, 22.4 and 35.8%, respectively. The EPDS negative group had more patients diagnosed with schizophrenia at 39.7%. When the EPDS scores were analyzed according to the diagnostic category, only the depressive disorders were statistically significant at 0.019. Most mothers received a secondary education (53.6%) and the largest group had a household income per month of < RM1000 (27.1%). Most mothers had secondary education at 53.6%. In the EPDS positive group, 6.0% of mothers had no formal education, compared to only 1.4% in the EPDS negative group. In this study, the mothers who earned at least RM3000 above had higher rates of EPDS positive. A quarter of mothers who had suffered domestic violence had EPDS positive, compared to 20.5% in those not exposed to domestic violence. A total of 47.8% of participants had postnatal depression. The overall mean EPDS score was 12.7 (7.2). The mean for positive EPDS score was 19.0 (4.0) and the mean for negative EPDS score was 7.0 (4.0). The mean age for the child was 10.1 (4.3) years. There were more girls in the EPDS positive mothers' group. Most children were from larger families with 3 or more siblings. The mean total SDQ score was 10.6 (6.3).

**Table 1:** Participants' demographics and clinical characteristics.

**Characteristics of participants and outcomes**		**Mean EPDS score (** ***n*** **)**			***P*-values**
		**Overall (*n*)**	**Negative (*n*)**	**Positive (*n*)**	
		**12.7 (*n* = 140)**	**7.0 (*n* = 73)**	**19.0 (*n* = 67)**	
Mean age in years (SD)		38.6 (7)	39.5 (6.5)	37.7 (7.4)	0.143
Ethnicity	Bajau (*n*)	11.7 (23)	6.7 (14)	19.4 (9)	0.484
	Bugis (*n*)	7.7 (3)	4.5 (2)	14.0 (1)	
	Chinese (*n*)	14.7 (18)	9.0 (8)	19.3 (10)	
	Kadazan (*n*)	13.6 (57)	8.0 (28)	19.0 (29)	
	Malay (*n*)	11.0 (9)	6.4 (5)	16.8 (4)	
	Murut (*n*)	10.0 (2)	0.00 (1)	20.0 (1)	
	Other (*n*)	11.8 (28)	5.2 (16)	19.3 (13)	
Religion	Agnostic (*n*)	18.0 (1)	0.0	18.0 (1)	0.595
	Buddhist (*n*)	16.0 (6)	9.7 (3)	22.3 (3)	
	Christian (*n*)	12.7 (58)	7.8 (31)	18.2 (27)	
	Islam (*n*)	12.4 (75)	6.1 (39)	19.3 (36)	
Marital status	Single (*n*)	11.4 (8)	7.4 (5)	18.0 (3)	0.831
	Married (*n*)	12.9 (114)	7.2 (57)	18.6 (57)	
	Divorcee/widow (*n*)	12.4 (18)	6.0 (12)	22.4 (7)	
Diagnosis	Anxiety disorders (*n*)	12.0 (17)	7.9 (10)	17.9 (7)	0.019
	Bipolar disorder (*n*)	13.2 (28)	6.5 (13)	18.9 (15)	
	Depressive disorders (*n*)	15.7 (38)	8.6 (14)	19.8 (24)	
	Schizophrenia (*n*)	11.0 (48)	6.7 (31)	18.2 (17)	
	Other (*n*)	9.7 (10)	3.3 (6)	19.3 (4)	
Education level	No formal education (*n*)	17.2 (5)	0.0 (1)	21.5 (4)	0.427
	Primary (*n*)	10.7 (11)	5.0 (6)	17.6 (5)	
	Secondary (*n*)	12.7 (75)	7.2 (40)	19.1 (35)	
	Tertiary (*n*)	12.7 (49)	7.5 (26)	18.7 (23)	
Household income per month	< RM1000 (*n*)	11.8 (38)	7.13 (24)	19.79 (14)	0.839
	RM1000–Rm2000 (*n*)	13 (35)	6.8 (16)	18.2 (19)	
	RM2001–Rm3000 (*n*)	12.7 (21)	7.0 (12)	20.2 (9)	
	RM3001–Rm4000 (*n*)	12.4 (16)	6.6 (8)	18.1(8)	
	More Than RM4000 (*n*)	13.8 (30)	7.2 (13)	18.9 (17)	
History of domestic violence	Yes (*n*)	14.6 (32)	8.1 (15)	20.3 (17)	0.100
	No (*n*)	12.2 (108)	6.7 (58)	18.5 (50)	
Age of child in years (SD)		10.1 (4.3)	10.5 (4.3)	9.7 (4.2)	0.242
Siblings	0 (*n*)	11.8 (16)	5.6 (9)	19.9 (7)	0.831
	1 (*n*)	12.4 (37)	8.7 (21)	17.1 (16)	
	2 (*n*)	12.3 (23)	5.3 (10)	17.7 (13)	
	3 and above (*n*)	13.3 (64)	6.8 (33)	20.3 (31)	
Gender of child	Male (*n*)	12.2 (74)	6.9 (42)	19.1 (32)	0.360
	Female (*n*)	13.3 (66)	7.1 (31)	19.0 (35)	
Total SDQ score (SD)		10.6 (6.3)	7.9 (5.4)	13.5 (6.0)	<0.001
SDQ emotional problems (SD)		2.4 (2.3)	1.5 (1.7)	3.4 (2.5)	<0.001
SDQ conduct problems (SD)		2.2 (1.8)	1.6 (1.5)	2.7 (1.8)	<0.001
SDQ hyperactivity (SD)		3.2 (2.3)	2.4 (2.3)	4.0 (2.1)	<0.001
SDQ peer problems (SD)		2.8 (2.0)	2.3 (1.8)	3.3 (2.0)	0.003
SDQ prosocial (SD)		7.5 (2.3)	7.8 (2.3)	7.2 (2.3)	0.169

Multiple linear regression models were used to determine which variables were associated with higher SDQ scores. Model 1 was unadjusted. Model 2 was adjusted for confounders namely maternal age, marital status, ethnicity, religion, maternal education, household income per month, history of domestic violence, maternal diagnosis, child age, child gender, and child's siblings. All analyses were performed using the Statistical Package for the Social Sciences (SPSS) version 26, licensed to UKM (44).

This study found that mothers who had postnatal depression were associated with higher total SDQ scores in their offspring, with an OR of 5.65 (3.74, 7.55) prior adjustment and an adjusted OR of 5.51 [95% CI (3.57, 7.46)] when adjusted for confounders. Looking into the subscales of the SDQ, the offspring also scored higher on emotional problems OR of 1.98 [95% CI (1.23, 2.72)], conduct problems 1.14 [95% CI (0.55, 1.72)], and hyperactivity subscale scores 1.47 [95% CI (0.74, 2.20)]. All above mentioned OR have a *p*-value of < 0.001. The only score which measures positive behavior was the prosocial score, which was lower when there was higher postnatal depression, OR of −0.56 [95% CI (−1.37, 0.25)], however *p*-value was not significant at 0.172 (Table 2). Our study reported an association between maternal postnatal depression and peer problems in their offspring with an OR of 0.94 [95% CI (0.26, 1.61)] with *p*-value of 0.007 (Table 2).

**Table 2:** Association of maternal postnatal depression with offspring psychological outcomes.

**Children psychological outcomes**	**Model 1**		**Model 2**	
	**Odds ratio (95% CI)**	***P*-values**	**Odds ratio (95% CI)**	***P*-values**
SDQ total difficulties	5.65 (3.74, 7.55)	<0.001	5.51 (3.57, 7.46)	<0.001
SDQ emotional problems	1.97 (1.26, 2.68)	<0.001	1.98 (1.23, 2.72)	<0.001
SDQ conduct problems	1.12 (0.55, 1.68)	<0.001	1.14 (0.55, 1.72)	<0.001
SDQ hyperactivity	1.59 (0.85, 2.33)	<0.001	1.47 (0.74, 2.20)	<0.001
SDQ peer problems	0.97 (0.33, 1.61)	0.003	0.94 (0.26, 1.61)	0.007
SDQ prosocial	−0.54 (−1.32 to 0.23)	0.169	−0.56 (−1.37 to 0.25)	0.172

*Model 1, Unadjusted; Model 2, Adjusted for maternal age, marital status, Ethnicity, religion, maternal education, household income per month, history of domestic violence, maternal diagnosis, child age, child gender, and child's siblings. P-value significant at < 0.05*.

## Discussion

This study is unique as it explores the rate of postnatal depression in the population of mental health clients. We found much higher rates of postnatal depression in mothers who are mental health clients than rates found in the community. This is not surprising, as existing mental health disorders predispose to another mental health disorder (11). It was found that postnatal depression is 20 times more likely if a person has had depression prior (45). However, a major limitation of this study was that it did not compare the mental health clients to a non-psychiatric group. Therefore, conditions specific to mental health clients could not be differentiated from those in the community.

The EPDS used was not initially designed to be a diagnostic scale, but further studies have shown that a cut off score of ≥13 indicates clinically significant depression (40). Although originally the EPDS was designed to assess postpartum depressive symptoms in the past 7 days postpartum, it has been found to be a valid measure of depressive symptomatology in parents beyond the immediate postpartum period (35). In the ALSPAC (37), a currently ongoing birth cohort, parents have been assessed using EPDS at time points up to the period of 11 years and planned to be assessed at 18 years because of the longitudinal nature of depression. The children reported in this study were about ten years old with the oldest being about 14 years old. It is not clear whether the depressive symptomatology reported were truly related to postpartum depression or more reflective of the mother's current state of mind. Interestingly, keeping in mind the longitudinal nature of depression, the relationship between these two distinct periods is still being studied (37).

Mothers who are in the lower socioeconomic strata are at higher risk of developing postnatal depression (46). This is important to note as more than 50 percent of the population of this study consisted of mothers whose families fell below the Malaysian poverty line. The Malaysian poverty line was reported to be RM 2208 in 2019 (47). This may partially account for such a high rate of postnatal depression in our study as well. There were a few findings which were contrary to current research. The household income also had a bearing in that, more affluent families had more rates of postnatal depression especially those who earned RM3000 or more per month. Higher socioeconomic status had been associated with less postnatal depression (46). Marriage was associated with a higher level of postnatal depression. The presence of a partner during pregnancy has been shown to be beneficial for a woman's mental health (48). Perhaps a larger sample size in future studies would approximate these findings.

Confounders in this study were maternal age, marital status, ethnicity, religion, maternal education, household income per month, history of domestic violence, concurrent maternal diagnosis, child age, child gender, and child's siblings. The findings could also be explained by a confounder not addressed in this study, which was the existing mental health diagnoses which could have given a false high result. In this study, when the EPDS scores were analyzed according to the diagnostic category, only the depressive disorders were statistically significant. This is consistent with previous studies which have found postnatal depression to be related to previous depression (45, 49, 50).In addition, this study did not differentiate between inpatients and outpatients. Inpatient and outpatient care signifies different illness severity, and conditions predisposing to severity of illness such as conditions and support at home, which may also provide variance to the findings.

Our study's findings reinforce previous studies results, that postnatal depression in mothers is positively associated with more difficulties in the psychological and emotional health of their children (51–54). The exception was for SDQ peer problems which were not echoed in other previous similar studies. While it is true that genetic inheritance plus the parenting behavior of a mother with mental illness would have an effect on the child, the mothers with depressive symptoms reported more behavior problems in their children compared to another group of mothers without depressive symptoms. However, one limitation in this study is that there is a possibility that the depressed mothers may have negatively rated their children due to processing bias. The EPDS is a self-rated tool with recall bias and it has been known to have low positive predictive values as found by a 2001 meta-analysis on EPDS (55). Therefore, it would have been more accurate to have an independent assessment of the children's behavior. A mother's depression would have also affected the other children in the family, but they were also not assessed.

We only examined correlations, and not causal relationships. Given the time constraints in this study, there was a relatively small recruitment of samples. Convenience sampling was used instead of random sampling. In this study, mothers were asked to rate postnatal depression in any pregnancy and the SDQ was also rated for any child. This meant that the postnatal depression symptoms may or may not have had a direct influence on the child with psychological issues.

Mental health services need to play a major role in the early detection of maternal depression and its prompt treatment in mental health clients. Mental health services need to be ready to intervene on the clients' children as well, given such a big impact on children. Early intervention can make a significant difference to the mother and subsequently positively affect the development of their children. A study found that intensive home-based treatment for maternal depression mitigated the negative effects of depression on children's development (56).

## Conclusion

This study highlights the importance of maternal mental health in influencing their children's health in turn. More focus needs to be given to maternal depression in mental health clients to improve mother and child well-being in the future. Mental health services need to play a major role in the early detection of maternal depression and its prompt treatment in mental health clients.

## Data Availability Statement

The datasets presented in this study can be found in online repositories. The names of the repository/repositories and accession number(s) can be found below: https://data.mendeley.com/datasets/hp6y7cj9r2/1.

## Ethics Statement

The studies involving human participants were reviewed and approved by UKM Research and Ethics Committee. Written informed consent to participate in this study was provided by the participants' legal guardian/next of kin.

## Author Contributions

FA came up with the initial idea, its execution plan, later reviewed drafts, and finalized this writing. YW and AK contributed to the data collection. YW carried out the statistical analyses and subsequently the initial draft. All authors contributed to the article and approved the submitted version.

## Conflict of Interest

The authors declare that the research was conducted in the absence of any commercial or financial relationships that could be construed as a potential conflict of interest.

## Publisher's Note

All claims expressed in this article are solely those of the authors and do not necessarily represent those of their affiliated organizations, or those of the publisher, the editors and the reviewers. Any product that may be evaluated in this article, or claim that may be made by its manufacturer, is not guaranteed or endorsed by the publisher.
